# Gastritis cystica profunda occurring under immunosuppression: A case report of a patient with autoimmune hepatitis

**DOI:** 10.1097/MD.0000000000049250

**Published:** 2026-06-12

**Authors:** Hong-Mei He, Shu-Yan Xu, Yi-Cun Liu, Han-Zhen Ji, Zhao-Lian Bian

**Affiliations:** a Department of Gastroenterology, Nantong Third People’s Hospital, Affiliated Nantong Hospital 3 of Nantong University, Nantong, Jiangsu, PR China; b Department of Library, Nantong Third People’s Hospital, Affiliated Nantong Hospital 3 of Nantong University, Nantong, Jiangsu, PR China.

**Keywords:** autoimmune hepatitis, case report, gastritis cystica profunda, immunosuppressant, ultrasound gastroscope

## Abstract

**Rationale:**

Gastritis cystica profunda (GCP) is a rare gastric submucosal lesion without typical clinical symptoms. Its pathogenesis has not yet been elucidated, and there is no consensus regarding its treatment, which primarily includes surgical and endoscopic methods. Autoimmune hepatitis (AIH) is an inflammatory liver condition characterized by elevated serum immunoglobulin G levels and multiple autoantibodies. Patients with AIH typically require long-term oral immunosuppressive therapy. However, whether immunosuppression accelerates or is correlated with the growth of gastric masses has not been reported.

**Patient concerns:**

A 51-year-old woman with AIH on long-term oral immunosuppression was found to have a gastric mass. Rapid growth of the mass was noted after more than 3 years of follow-up, and pathology was suggestive of GCP after endoscopic submucosal dissection.

**Diagnoses:**

We report the gastroscopic, ultrasonographic, and computed tomography manifestations, as well as the diagnostic and therapeutic procedures, of a case of GCP in a patient with AIH on long-term oral immunosuppression, with the aim of improving clinicians’ knowledge of this disease.

**Interventions:**

As endoscopic ultrasound indicated that the lesion was localized within the mucosal layer, beneath which a tubular anechoic area was observed, we performed endoscopic submucosal dissection to achieve en bloc resection of the lesion.

**Outcomes:**

Postoperative follow-up revealed that the patient recovered well. The patient in this case report had an incidental finding of gastric body GCP with no previous history of gastric surgery but had a clear history of autoimmune disease and immunosuppressant administration. Therefore, we speculate that GCP may be correlated with immune modulation. Additionally, immunosuppressive agents may contribute to the development of GCP.

**Lessons:**

Although the pathogenesis of GCP remains unclear, this case suggests that long-term immunosuppressive therapy may accelerate its progression.

## 1. Introduction

Gastritis cystica profunda (GCP), also known as polypoid cystic gastritis, is a rare submucosal lesion characterized by the growth of gastric mucosal glands in the layer below the muscularis mucosa and the formation of cystic dilatations.^[1–3]^ The pathogenesis of GCP remains unclear, although previous studies have suggested that it is induced by invasive gastric procedures such as gastric surgery, biopsy, and polypectomy.^[4]^ However, recent studies have shown that many patients with GCP have no history of surgery or invasive gastric procedures. Chronic active and atrophic gastritis may be another significant contributing factor in the development of GCP.^[5–7]^

Owing to the atypical clinical manifestations and endoscopic features of GCP,^[3,8]^ misdiagnoses and missed diagnoses are often encountered in clinical practice. Endoscopic manifestations of GCP can be divided into 4 main types: submucosal eminence, gastric mucosal hypertrophy, gastric mucosal lesions, and polypoid eminence. Of these, submucosal eminence is the most common, accounting for approximately 45%, and its typical changes on endoscopic ultrasonography (EUS) are hypoechoic or tube-like anechoic cystic changes in the submucosa of the stomach.^[9]^ On imaging, GCP typically presents as round or oval gastric wall masses with diameters of <5 cm. The lesions exhibit computed tomography (CT) attenuation values lower than those of soft tissue, approximating the density of water. Furthermore, contrast-enhanced CT demonstrates marked enhancement of the lesion surface with well-defined margins, frequently accompanied by “sandwich sign” enhancement or “honeycomb sign” patterns. These features likely result from gastric mucosal hyperplasia and inflammatory responses.^[10,11]^ Wang et al found that submucosal cystic changes and delayed enhancement of peripheral marginal bands may be valuable CT features for differentiating GCP from other lesions.^[2,12]^ The pathological diagnostic criteria for GCP are cystically dilated glands in the submucosa or muscularis propria layer, accompanied by connective tissue hyperplasia and chronic inflammatory cell infiltration.^[13]^ GCP primarily requires differentiation from the following entities: gastric stromal tumors (appearing as solid masses on EUS), gastric hamartomas (often associated with hereditary syndromes and lacking peripheral rim enhancement), gastric adenocarcinoma (exhibiting dysplasia on pathology), gastric leiomyomas (homogeneous hypoechoic masses arising from the muscularis propria on EUS), giant hypertrophic gastritis (Menetrier disease, predominantly involving the mucosal layer), and ectopic pancreas (characterized by the central umbilication sign).^[12]^

Autoimmune hepatitis (AIH) is an inflammatory liver disorder characterized by elevated serum immunoglobulin G levels and the presence of multiple autoantibodies.^[14,15]^ When first-line therapy yields suboptimal responses, adjunctive immunosuppressive agents such as mycophenolate mofetil (MMF) should be incorporated into the treatment regimen.^[15,16]^ The most common gastrointestinal side effects of MMF include nausea, diarrhea, vomiting, ulceration, abdominal pain, and rare gastrointestinal bleeding. Furthermore, MMF carries a 1.83-fold increased risk of gastroduodenal erosions.^[17]^ Notably, Moutsoglou et al reported a case of cystic lesion development in the transverse colon secondary to MMF administration.^[18]^ This finding confirms that immunosuppressive agents may not only induce gastrointestinal side effects but also trigger subsequent gastrointestinal pathologies. To date, there have been no reported cases of immunosuppressant-induced GCP. This article presents a case from the Department of Gastroenterology, Nantong Third People’s Hospital, detailing a patient with AIH on long-term MMF therapy who was diagnosed with GCP during gastroscopic examination. The report aims to provide novel insights into GCP pathogenesis and enhance clinical vigilance regarding this disorder.

## 2. Case presentation

**Figure 1.**
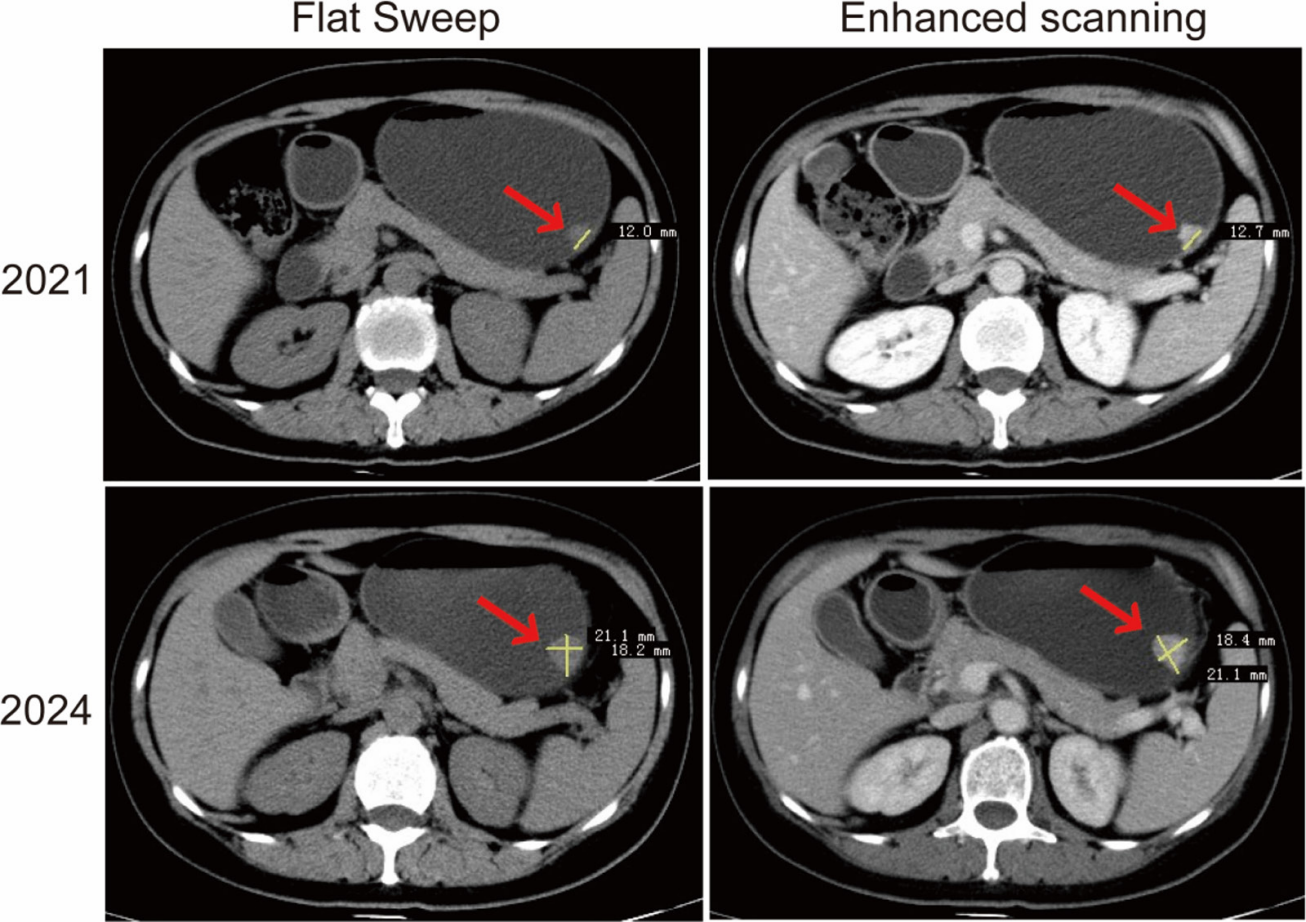
Computed tomography images from 2021 to 2024.

**Figure 2.**
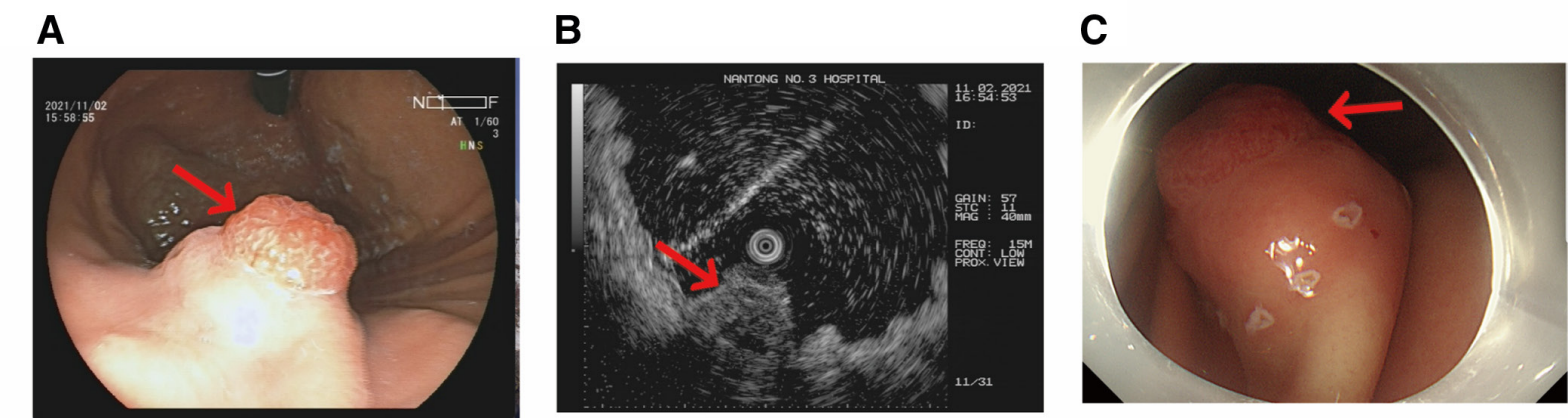
Image of GCP lesion gastroscopy. (A) Gastroscopy revealed a bulging lesion on the side of the greater curvature of the gastric body. (B) An ultrasonographic image from 2021 indicated that the lesion was located in the mucosal layer, and a duct-like echogenic area was observed underneath. (C) Marking of the lesional margins. GCP = gastritis cystica profunda.

**Figure 3.**
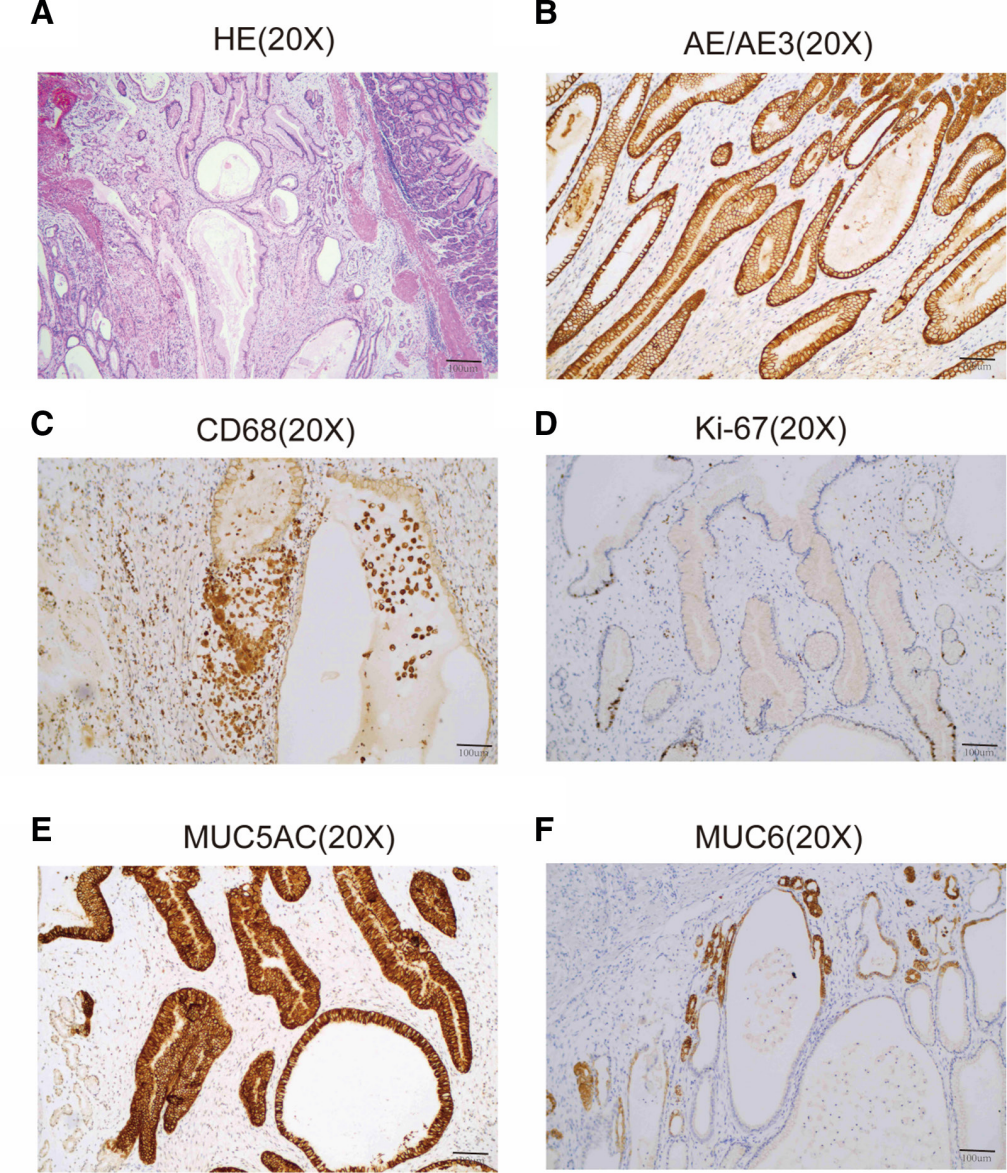
Histopathological staining. (A) Hematoxylin and eosin staining indicated dilatation of the submucosal glands. (B–F) Immunohistochemical staining: (B) AE/AE3, (C) CD68 (D) Ki-67, (E) MUC5AC, and (F) MUC6. AE/AE3 = anti-cytokeratin AE/AE3, CD68 = cluster of differentiation 68, Ki-67 = Kiel-67, MUC5AC = Mucin 5AC, MUC6 = Mucin 6.

A 51-year-old woman was admitted to the hospital with a gastric mass that had been present for more than 3 years. The patient had a history of AIH for more than 4 years and had been taking oral MMF tablets (CellCept; Roche Pharmaceuticals) and ursodeoxycholic acid capsules (Ursofalk; Dr. Falk Pharma GmbH, Germany) for an extended period. A gastroscopic examination performed in November 2021 revealed a gastric polyp measuring approximately 1.0 cm in the greater curvature of the gastric body, with a flush surface and a possible opening in the center (Fig. 2A). Contrast-enhanced CT revealed a lesion in the greater curvature of the gastric body, which was considered a possible ectopic pancreas (Fig. 1). EUS indicated that the lesion was in the mucosal layer, beneath which a tubular anechoic area was observed (Fig. 2B). Pathological biopsy revealed that the lesion was likely a hyperplastic polyp. The patient was advised to undergo gastroscopic reexamination 3 to 6 months later to determine the next course of treatment. In September 2022, follow-up EUS showed a 1.0 cm protrusion on the greater curvature of the gastric body. The lesion originated from the muscularis propria layer with an isoechoic pattern, beneath which a tubular hypoechoic pattern was detected. The patient decided to undergo regular follow-up examinations rather than endoscopic treatment. In April 2024, enhanced abdominal CT revealed a lesion on the greater curvature of the stomach that was suspected to be a stromal tumor, approximately 2.1 × 1.8 cm^2^ in size, which was significantly larger than it had been previously (Fig. 1). Additional laboratory examinations included measurement of Ig (IgG, IgM, and IgA) and AIH-associated serologic marker levels, as well as screening for associated viral infections, including Epstein–Barr virus (EBV) and cytomegalovirus. The erythrocyte sedimentation rate was 40 mm/h. However, routine laboratory assessments, including blood panels, hepatorenal function tests, coagulation profiles, and tumor marker analyses, revealed no further clinically significant abnormalities (Table 1). With the consent of the patient and her family, endoscopic submucosal dissection (ESD) was performed to remove the lesion, mark the lesion margins, and completely excise the 2.3 × 2 × 1 cm^3^ specimen (Fig. 2C); no postoperative bleeding or perforation occurred. Postoperative pathological examination revealed a neoplasm in the gastric body, exhibiting features consistent with GCP (Fig. 3A). Immunohistochemical analysis of paraffin block B demonstrated anti-cytokeratin AE1/AE3 positivity (Fig. 3B), the presence of cluster of differentiation 68-positive histiocytes (Fig. 3C), desmin-positive smooth muscle cells, Kiel-67 (Ki-67) positivity (Fig. 3D), and focal expression of Mucin 5AC (Fig. 3E) and Mucin 6 (Fig. 3F), whereas cluster of differentiation 10, Mucin 2, and tumor protein p53 (P53) were negative. The specimen had an intact perimembrane and negative lateral and basal margins. Postoperative follow-up revealed that the patient had recovered well.

**Table 1:** General clinical data.

	Variant	Value
Biochemical indicators	ALT (U/L)	12
	AST (U/L)	18
	ALB (g/L)	44.9
	BUN (mmol/L)	3.76
	Cr (µmol/L)	59.9
	K^+^ (mmol/L)	3.68
	Na^+^ (mmol/L)	138.7
	PT (s)	12.1
	CEA (ng/mL)	0.59
Immunological indicators	IgG (g/L)	13.00
	IgM (g/L)	1.40
	IgA (g/L)	2.22
	ANA (<1:80)	1:00
	SMA (negative)	(−)
	AMA (negative)	(−)
Viral infections	EB-DNA (<5.0 × 10^2^ IU/mL)	<5.0 × 10^2^
	CMV-DNA (<5.0 × 10^2^ IU/mL)	<5.0 × 10^2^

ALB = albumin, ALT = alanine aminotransferase, AMA = anti-mitochondrial antibody, ANA = antinuclear antibody, AST = aspartate aminotransferase, BUN = blood urea nitrogen, CEA = carcinoembryonic antigen, CMV-DNA = cytomegalovirus DNA, Cr = creatinine, EB-DNA = Epstein–Barr virus DNA, IgG/M/A = immunoglobulin G/M/A, PT = prothrombin time, SMA = smooth muscle antibody.

## 3. Discussion

The etiology and pathogenesis of GCP remain unclear, and its clinical manifestations lack specificity. There are only a few individual case reports and no multicenter clinical studies on GCP with large sample sizes, either nationally or internationally. There are currently no guidelines for the diagnosis and treatment of GCP. Du et al retrospectively collected and analyzed the clinical data of 57 patients with GCP. The main clinical manifestation was abdominal pain (n = 14, 36.8%), and 28.9% (n = 11) of the patients had no significant clinical symptoms. The most common anatomical location of GCP was the gastric body (n = 30, 58.8%).^[3]^ The patient in the present case report had an incidental finding of gastric body GCP with no previous history of gastric surgery but had a clear history of autoimmune disease and immunosuppressant administration. Therefore, we speculate that GCP may be correlated with human immunomodulation. The immunological mechanism of MMF-induced gastritis primarily stems from the dual damaging effects of the active metabolite, mycophenolic acid (MPA), on the gastrointestinal mucosa, as it directly inhibits epithelial cell proliferation while disrupting mucosal immune homeostasis.^[19]^ First, MPA directly induces epithelial cell apoptosis, compromising gastric mucosal integrity and triggering inflammatory responses. Second, MPA selectively inhibits inosine monophosphate dehydrogenase, an enzyme critical for maintaining regulatory T cell suppressive functions. By reducing regulatory T cell numbers and functionality, MPA promotes the release of pro-inflammatory cytokines (e.g., tumor necrosis factor-α and interleukin-1β), ultimately driving mucosal inflammation.^[20,21]^ Currently, there are no reported associations between MMF and GCP, and the specific molecular mechanisms require further validation through clinical and fundamental experiments.

EUS revealed a submucosal anechoic area in our patient with GCP. The patient’s 2024 CT scan revealed a prominent oval cystic change in the submucosa, with CT values within the lesion slightly lower than those of water. Contrast-enhanced CT revealed delayed enhancement of the peripheral rim of the lesion. Furthermore, hematoxylin and eosin staining indicated significant dilation of the glands in the submucosa. Immunostaining for the gastric fundic gland polyp markers revealed focal expression of Mucin 5AC and Mucin 6. Based on EUS, CT imaging, and pathology findings, the patient was diagnosed with GCP. Conventional studies have regarded GCP as a premalignant lesion with the potential for malignant progression.^[22,23]^ However, a recent clinical study involving 1432 patients (including 180 in the GCP group) showed that all gastric cancers were in the mucosal layer, with no cases arising within the GCP itself. This suggests that GCP is a paralesional lesion that indirectly contributes to tumorigenesis by inducing chronic gastric mucosal injury.^[13]^ Furthermore, 13% of gastric cancer cases are associated with GCP, with 60.2% of the tumors located adjacent to or overlying GCP lesions. The GCP group showed significantly elevated rates of P53 positivity, Ki-67 expression, EBV infection, and programmed death-ligand 1 composite scores. These findings confirmed the pathological association between gastric carcinogenesis and GCP.^[13]^ Studies have indicated a reciprocal inhibitory relationship between P53 and inflammation, whereas EBV triggers inflammatory responses.^[24]^ This suggests that the underlying GCP-induced gastric pathologies (e.g., chronic gastritis and ulcers) may modulate P53 and EBV activity, thereby increasing the risk of cancer. Histopathological examination revealed Ki-67 positivity, indicating potential malignant progression of the GCP lesion. The patient was advised to undergo surveillance gastroscopy at our institution after 6 months, which demonstrated no significant abnormalities.

Current treatment options for GCP include endoscopic resection and surgical interventions. Endoscopic resection, including endoscopic mucosal resection and ESD, is the first choice for diagnosing GCP in the absence of other malignant lesions.^[25]^ Xu et al found that the complete resection rate of ESD (92%) was significantly higher than that of endoscopic mucosal resection (78%), suggesting that ESD is more suitable for the endoscopic treatment of GCP.^[10]^ Compared with surgery, endoscopic resection offers greater safety, faster recovery, and lower cost, although it may require a longer hospital stay. Most importantly, it maximizes the preservation of gastric function. However, with an increasing number of reports of gastric cancer combined with GCP,^[2,26,27]^ surgical resection should be the treatment choice for patients with GCP whose biopsy pathology suggests a potential malignancy.^[8]^ Surgical resection includes local tumor resection, partial gastrectomy, and total gastrectomy.

The present study had several limitations. First, the limited number of GCP cases at our center precluded comparative analysis with other cohorts. Second, suboptimal documentation of endoscopic images and specimen photography resulted in insufficient quality of the available visual data for detailed assessment.

## 4. Conclusion

GCP is a rare gastric disease with atypical clinical manifestations. CT findings suggestive of submucosal cystic changes and delayed enhancement of the peripheral marginal bands are highly suggestive of GCP. The combination of ultrasound gastroscopy and lesion biopsy can assist in diagnosis and planning. Furthermore, although a growing body of research suggests that GCP is not a precancerous lesion, it may mediate underlying gastric mucosal pathologies, thereby indirectly increasing the risk of tumorigenesis. Our case highlights the potential correlation between GCP and host immune mechanisms, suggesting a novel direction for investigating GCP pathogenesis. The specific mechanisms of interaction between GCP and the human immune system require further validation through large-sample multicenter clinical and basic research studies.

## Author contributions

**Conceptualization:** Zhao-Lian Bian.

**Methodology:** Shu-Yan Xu, Han-Zhen Ji.

**Data curation:** Han-Zhen Ji.

**Project administration:** Shu-Yan Xu.

**Formal analysis:** Yi-Cun Liu.

**Funding acquisition:** Yi-Cun Liu.

**Investigation:** Yi-Cun Liu.

**Supervision:** Zhao-Lian Bian.

**Visualization:** Hong-Mei He.

**Writing – original draft:** Hong-Mei He.

**Writing – review & editing:** Hong-Mei He.
